# Bilateral Stellate Ganglion Block After Ventricular Arrhythmia May Hide Clinical Signs of the Cushing Triad: A Case Study and Review of the Literature

**DOI:** 10.1155/crcc/6360234

**Published:** 2026-05-26

**Authors:** Michael Watchi, Victor Penaud, Valentina Ditali, Fabio Silvio Taccone

**Affiliations:** ^1^ Department of Intensive Care Medicine, Hôpital Universitaire de Bruxelles, Brussels, Belgium; ^2^ Médecine Intensive Réanimation, CHU Ambroise Paré, Assistance Publique des Hôpitaux de Paris, Paris, France

**Keywords:** Cushing reflex, intracranial hypertension, stellate ganglion, ventricular arrhythmia

## Abstract

**Background:**

Stellate ganglion blockade (SGB) has been described as a rescue therapy in recurrent electrical storm, with very few reported complications, except local hematomas. Yet the proximity of major blood vessels and the risk of a sympathetic reaction could expose patients to additional adverse events.

**Case Report:**

We reported the case of a 34‐year‐old patient, in whom a bilateral SGB was performed after in‐hospital cardiac arrest due to recurrent polymorphic ventricular tachycardia. She developed bilateral areflexic mydriasis, diffuse cerebral edema, pneumocephalus, and brainstem herniation and ultimately died from acute intracranial hypertension. We suspected that bilateral SGB, with its most frequent known complications, Horner′s syndrome, might have hidden neurological worsening by blunting some clinical signs, which resulted in delayed diagnosis of intracranial hypertension and brain swelling.

**Conclusions:**

The blocking of sympathetic efferences of the stellate ganglion may result in transient effects that interfere with neurological examination, especially when performed bilaterally.

## 1. Introduction

Stellate ganglion blockade (SGB) has been recently described as a rescue therapy in refractory ventricular arrhythmias, which were evolving to electrical storm (ES) [1]. By modulating sympathetic outflow, SGB may reduce arrhythmia burden and decrease recurrence rates when conventional antiarrhythmic therapies are insufficient. Reported clinical experience suggests a favorable safety profile, with most complications being infrequent and generally manageable. Rare but serious adverse events have been described, including airway compromise due to cervical hematoma and infectious complications leading to neurological deficit [2]. Overall, available data indicate that the procedural risk remains low, even in patients receiving anticoagulant therapy [3].

After obtaining informed consent from the patient′s legal representative, we reported the case of a bilateral SGB performed following return of spontaneous circulation (ROSC) after an in‐hospital cardiac arrest, presumed to be secondary to recurrent polymorphic ventricular tachycardia. The well‐recognized and typically transient effects of SGB on pupillary function may have contributed to a delay in the recognition of acute intracranial hypertension following cardiac arrest.

## 2. Clinical Case

A 34‐year‐old female patient was admitted to the intensive care unit (ICU) following an in‐hospital cardiac arrest. Her medical history was notable for a left knee chondrosarcoma diagnosed in 2008, complicated with lung metastases, including a lesion located beneath the tracheal carina. During the 3 weeks preceding the event, she had been hospitalized in the oncology ward for treatment of bacteremia caused by *Enterococcus faecium* and *Veillonella parvula*; she was receiving intravenous vancomycin, piperacillin–tazobactam, and doxycycline. Despite a comprehensive evaluation, including physical examination and thoracoabdominal computed tomography (CT), no clear infectious source was identified.

**Figure 1:**
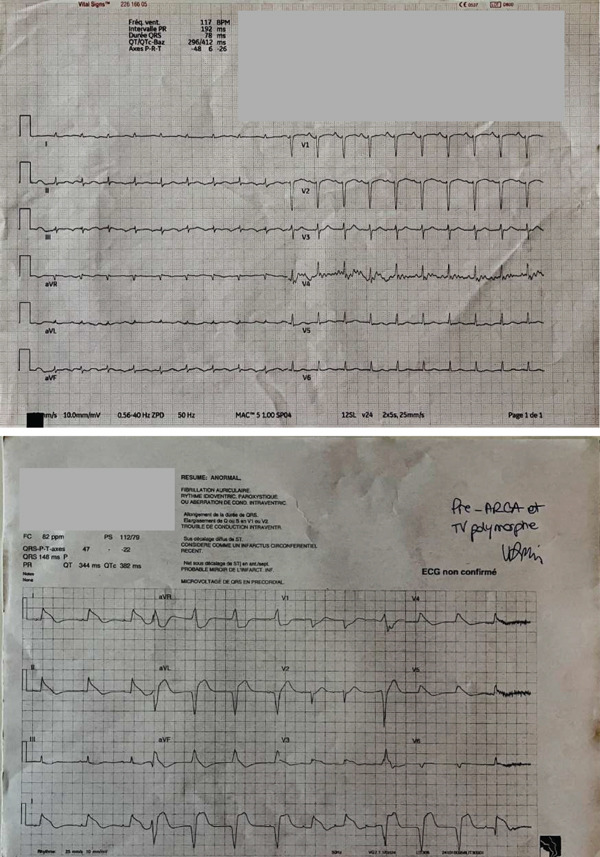
Of importance electrocardiography (ECG) drawn during her ICU stay. The upper ECG has been drawn after obtaining stable ROSC following her first cardiac arrest. It shows sinus rhythm, normal PR interval (192 ms), normal QRS length (78 ms), normal QRS axis, normal ST segment, and normal QT (412 ms with the Bazett formula). The under ECG has been drawn shortly after recovering a stable ROSC during the second episode of cardiac arrest and shows a polymorphic ventricular tachycardia.

While hospitalized, the patient experienced a witnessed cardiac arrest associated with ventricular fibrillation. No‐flow time was 0 min, and low‐flow time was 9 min; stable ROSC was achieved after three external defibrillation shocks and administration of intravenous amiodarone (300 mg). She was subsequently transferred to the ICU for postcardiac arrest care. Sedation was rapidly discontinued, and the patient was extubated without complication with a normal neurological examination. No clear etiology for the cardiac arrest was identified. Postresuscitation electrocardiography showed no conduction or repolarization abnormalities, and transthoracic echocardiography was unremarkable. Coronary CT angiography demonstrated no significant calcium deposition, making obstructive coronary artery disease unlikely. Comprehensive thoracoabdominal and cerebral CT imaging revealed no acute abnormalities. Laboratory investigations remained within normal limits. As she required no vasopressor or oxygen support and reported no symptoms, no antiarrhythmic therapy was initiated following consultation with the institutional electrophysiology team. The electrocardiogram after hemodynamic stabilization is shown in Figure 1.

Two days after ICU admission, she experienced a second in‐hospital cardiac arrest. The event was preceded by an abrupt loss of consciousness, bradycardia, and hypotension. Her rhythm rapidly deteriorated into polymorphic ventricular tachycardia (Figure 1). Her airway was secured immediately, and a total of five external defibrillation shocks and a cumulative dose of 450 mg of amiodarone were administered. No epinephrine was given; stable ROSC was achieved after 12 min of resuscitation. In accordance with the cardiology team, bilateral SGB was performed as a rescue intervention. Under ultrasound guidance, a total of 10 mg of lidocaine and 20 mg of ropivacaine were administered locally.

**Figure 2:**
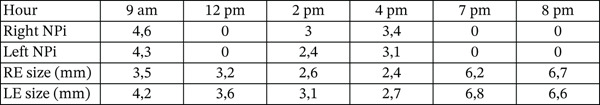
Automated pupillometry values over time. RE: right eye; LE: left eye, NPi: Neurological Pupil index.

**Figure 3:**
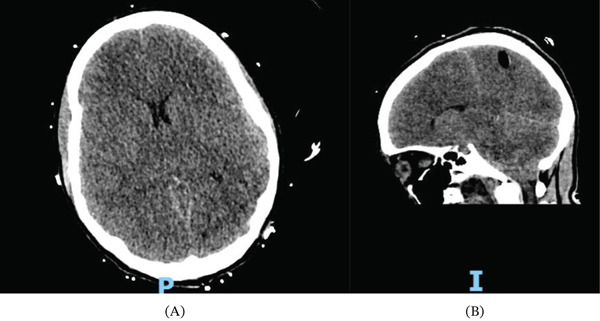
Cerebral CT imaging after the disclosure of bilateral areflexic mydriasis showing pneumocephalus and diffused cerebral edema. (A) Axial view and (B) coronal view (P: posterior; I: inferior).

Postresuscitation management included sedation with continuous infusions of propofol and sufentanil. Continuous electroencephalographic (EEG) monitoring was initiated, and blood cultures and routine laboratory tests were obtained. Immediately after hemodynamic stabilization, transcranial Doppler (TCD) examination showed values within normal range, with pulsatility indices (PIs) of approximately 0.8 and end‐diastolic velocities around 25 cm/s in both middle cerebral arteries. Automatic quantitative pupillometry (NPi‐300 Pupillometer, NeurOptics, Irvine, United States) performed shortly after bilateral SGB demonstrated bilateral nonreactive pupils without miosis or anisocoria. Pupillary reactivity recovered rapidly. Eight hours after the second cardiac arrest, pupillometry revealed abrupt bilateral areflexic mydriasis (Figure 2). Concurrently, continuous EEG monitoring demonstrated marked deterioration, evolving from severe encephalopathy with preserved background activity to electrocerebral silence. Urgent cerebral CT scan angiography (Figure 3) demonstrated diffuse cerebral edema, signs of intracranial hypertension, pneumocephalus, brainstem herniation, and globally reduced cerebral perfusion. Transesophageal echocardiography with contrast excluded a patent foramen ovale. Bilateral cervical showed no residual air where SGB had been performed.

**Figure 4:**
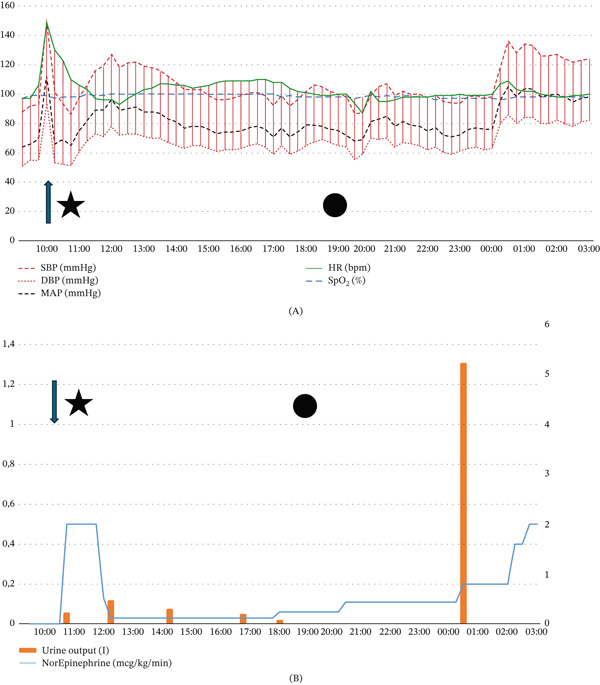
Patient′s hemodynamic parameters, with vasopressor and sedation use. (A) The patient′s physiological parameters over time and (B) changes in vasopressor requirements and urine output. The arrow marks the occurrence of the second cardiac arrest. The star indicates the time at which the bilateral stellate ganglion block (SGB) was performed. The circle denotes the onset of bilateral areflexic mydriasis. The *x*‐axis in both panels represents time in hours. In Figure 4B, the left *y*‐axis corresponds to norepinephrine infusion rate (micrograms per kilogram per minute), and the right *y*‐axis represents urine output (liters). SBP: systolic blood pressure; DBP: diastolic blood pressure; MAP: mean arterial pressure; HR: heart rate; SpO_2_: peripheral oxygen saturation.

Given the extent of diffuse cerebral edema and brainstem involvement, the multidisciplinary ICU team determined that the likelihood of meaningful neurological recovery was negligible. After discussion, life‐sustaining therapies were withdrawn, and the patient subsequently died. Consent for autopsy could not be obtained. A review of hemodynamic parameters from ROSC until the development of bilateral fixed mydriasis revealed a stable heart rate and rhythm, ranging between 60 and 110 beats per minute without significant variability. (Figure 4). Following the second cardiac arrest, norepinephrine infusion was initiated to maintain a mean arterial pressure above 65 mmHg with moderate doses (i.e., from 0.5 to 0.03 *μ*g/kg·min). Importantly, from the time intracranial hypertension was diagnosed until withdrawal of life‐sustaining therapy, no significant alterations in heart rate or rhythm were observed.

## 3. Discussion

ES is generally defined as recurrent episodes of sustained monomorphic or polymorphic ventricular tachycardia occurring within a short time frame. Although some authors propose specific numerical thresholds, such as three or more episodes within 24 h, there is no universally accepted definition [4, 5]. Over the past decade, SGB has emerged as a therapeutic option in the management of refractory ES. The role of sympathetic activation in the initiation and maintenance of ventricular arrhythmias is well established, and excessive adrenergic stimulation may facilitate progression to ES. Surgical sympathetic denervation has long been described as a treatment strategy in this context; however, it has significant complications [3, 5, 6]. Percutaneous SGB represents a less invasive alternative, as it can be performed at the bedside under ultrasound guidance, does not require transfer of hemodynamically unstable patients, and can be implemented rapidly without the logistical preparation associated with surgical procedures. This approach may provide a therapeutic window, allowing time for etiological investigation and planning of definitive interventions such as catheter ablation or device therapy [3–7].

The stellate ganglion, formed by fusion of the inferior cervical and first thoracic sympathetic ganglia, is a key component of the cervical sympathetic chain; it provides sympathetic innervation to the head, neck, heart, and upper extremities [8, 9]. Cardiac sympathetic fibers originating from the stellate ganglion connect to the central nervous system and modulate myocardial excitability. Importantly, functional lateralization exists: The left stellate ganglion predominantly influences ventricular myocardium, whereas the right stellate ganglion exerts greater modulation of sinoatrial node activity [7, 10]. Because ventricular arrhythmias are more closely linked to left‐sided sympathetic output, left SGB is generally preferred in cases of ventricular tachyarrhythmia. However, unilateral blockade may be insufficient, and repeated or bilateral SGB has been described in refractory ES.

SGB is generally considered safe, particularly when performed under ultrasound guidance. The most common expected effect is ipsilateral Horner′s syndrome, characterized by ptosis, miosis, and anhidrosis. Other reported complications are summarized in Table 1 [2, 4, 11–14]. Even in high‐risk populations, including patients with severe cardiomyopathy, cardiogenic shock, implanted defibrillators, or septic shock, SGB has demonstrated an acceptable safety profile. In a recent multicenter study, bilateral SGB was typically performed in sedated patients to mitigate the risk of respiratory compromise related to potential recurrent laryngeal or phrenic nerve involvement from local anesthetic spread [11, 14]. The duration of sympathetic blockade depends on the pharmacokinetics of the local anesthetic used. Lidocaine, bupivacaine, and ropivacaine are most frequently administered. Combining a short‐acting agent with a longer acting agent facilitates rapid onset and prolonged blockade (Table 2) [15]. Although the pharmacological effect of local anesthetics typically lasts several hours, reductions in arrhythmic burden have been reported for up to 72 h following SGB [11, 12, 14]. Notably, the clinical signs of Horner′s syndrome may resolve before complete recovery of sympathetic function [16].

**Table 1:** Reported complications and side effects of stellate ganglion block.

Neuraxial, phrenic nerve, or brachial plexus spread of local anesthetic
Hoarseness, dysphonia, brachial plexus block, transient Horner syndrome, anisocoria
Hemodynamic
Hypertension or hypotension, bradycardia
Vascular puncture
Neural puncture
Pneumothorax
Thyroid injury
Esophageal and tracheal puncture
Intravascular injection
Neck pain
Infections

**Table 2:** Pharmacokinetics of commonly used local anesthetics (LAs) for SGB.

Type of LA	Onset	Duration (minutes)	Cardiac toxicity
Lidocaine	Short	60–120	At high doses
Bupivacaine	Prolonged	180–420	Risky
Ropivacaine	Prolonged	180–420	Extremely low

In the present case, recurrent polymorphic ventricular tachycardia led to two cardiac arrests within 24 h. Following the first episode, the patient achieved full neurological recovery, and extensive investigations, including electrocardiography, transthoracic echocardiography, laboratory testing, whole‐body CT imaging, and coronary imaging, failed to identify a precipitating cause. Prior to the second event, she was awake, extubated, hemodynamically stable, and did not require vasopressor or inotropic support. The second arrest was again precipitated by polymorphic ventricular tachycardia. After successful defibrillation and amiodarone administration, bilateral SGB was performed to reduce arrhythmic recurrence and allow further evaluation, including consideration of electrophysiological testing. Bradycardia preceding ventricular tachycardia supported the rationale for bilateral sympathetic modulation. The patient was therefore sedated and mechanically ventilated; immediately after SGB, pupillary reactivity was reduced, and pupils appeared bilaterally small and poorly reactive, consistent with bilateral sympathetic blockade. TCD performed at that time showed normal flow velocities and PIs in both middle cerebral arteries, and intracranial hypertension was not suspected [17]. Over subsequent hours, pupillary reactivity transiently improved without recurrence of ventricular arrhythmias. Moreover, 8 h after SGB, the patient developed bilateral areflexic mydriasis. CT imaging demonstrated diffuse cerebral edema with herniation. Continuous EEG showed abrupt progression to electrocerebral silence.

We hypothesized that the longer half‐life of ropivacaine compared with lidocaine may have contributed to delayed and evolving autonomic effects [15]. During this period, hemodynamic parameters remained stable, suggesting continued sympathetic blockade. Approximately 5 h after the onset of bilateral mydriasis, the patient developed polyuria without concurrent bradycardia, hypertension, or abrupt vasopressor changes. This was interpreted as possible central diabetes insipidus in the context of catastrophic intracranial hypertension. Hemodynamic instability subsequently developed, accompanied by escalating vasopressor requirements, consistent with progression toward brain death. Retrospective review suggested that bilateral SGB may have attenuated the expected cardiovascular manifestations of Cushing′s response, potentially delaying clinical suspicion of intracranial hypertension.

Cushing′s triad, that is, hypertension, bradycardia, and abnormal respiratory pattern, is classically associated with rising intracranial pressure and cerebral circulatory arrest. This reflex represents a compensatory sympathetic surge aimed at preserving cerebral perfusion pressure. Although widely described, its exact incidence remains uncertain. Arrhythmias occur in approximately one‐third of patients progressing to brain death, and hypotension and polyuria are common in advanced stages [18, 19]. The Cushing response is often biphasic: initial sympathetic activation with tachycardia and hypertension, followed by sustained hypertension and reflex bradycardia; brainstem compression may produce diverse arrhythmias [20, 21]. By pharmacologically interrupting bilateral stellate ganglia, sympathetic efferent pathways to the heart may have been blunted, potentially masking the catecholamine surge typically observed during intracranial hypertension. This autonomic modulation could explain the absence of classic hemodynamic warning signs in this case.

Following neurological deterioration, comprehensive investigations were undertaken. Cervical ultrasound excluded local complications of SGB such as hematoma or residual air. Thoracoabdominal CT did not reveal systemic air embolism or pulmonary embolism. Transesophageal echocardiography with contrast excluded intracardiac shunt. No ventilator‐related barotrauma was identified. In retrospect, earlier repetition of TCD and automated pupillometry as part of a structured multimodal neurological monitoring strategy may have facilitated earlier detection of evolving intracranial hypertension and prompted earlier neuroimaging [22, 23].

## 4. Conclusions

SGB has recently gained attention as a rescue intervention for recurrent ventricular arrhythmias and ES. Bilateral SGB is less commonly employed and remains less extensively described in the literature. To our knowledge, this is the first report suggesting a potential association between bilateral SGB and delayed recognition of intracranial hypertension due to masking of neurological warning signs. We therefore suggest that when bilateral SGB is performed, particularly in patients who remain sedated, neurological multimodal monitoring should be used.

## Funding

No funding was received for this manuscript.

## Conflicts of Interest

None of the authors have a conflict of interest to disclose.

## Data Availability

The data that support the findings of this study are available on request from the corresponding author. The data are not publicly available due to privacy or ethical restrictions.
